# Neuroinflammatory Biomarkers in Alzheimer’s Disease: From Pathophysiology to Clinical Implications

**DOI:** 10.3390/ijms252211941

**Published:** 2024-11-06

**Authors:** Fausto Roveta, Lucrezia Bonino, Elisa Maria Piella, Innocenzo Rainero, Elisa Rubino

**Affiliations:** Aging Brain and Memory Clinic, Department of Neuroscience “Rita Levi-Montalcini”, University of Torino, 10126 Torino, Italy; fausto.roveta@unito.it (F.R.); lucrezia.bonino@unito.it (L.B.); elisamaria.piella@unito.it (E.M.P.); innocenzo.rainero@unito.it (I.R.)

**Keywords:** Alzheimer’s disease, neuroinflammation, biomarkers, neurobiological diseases, neuropathogenesis

## Abstract

The identification of neuroinflammation as a critical factor in Alzheimer’s disease (AD) has expanded the focus of research beyond amyloid-β and tau pathology. The neuroinflammatory fluid biomarkers GFAP, sTREM2, and YKL-40 have gained attention for their potential in early detection and monitoring of disease progression. Plasma GFAP has demonstrated promise in predicting the conversion from mild cognitive impairment to AD dementia, while sTREM2 highlights microglial activation, although there are conflicting results regarding its dynamics in AD pathogenesis. Advanced imaging techniques, such as PET tracers targeting TSPO and MAO-B, have also been developed to visualize glial activation in vivo, offering spatial and temporal insights into neuroinflammatory processes. However, the clinical implementation of these biomarkers faces challenges due to their lack of specificity, as many of them can be elevated in other conditions. Therapeutic strategies targeting neuroinflammation are emerging, with TREM2-targeting therapies and antidiabetic drugs like GLP-1 receptor agonists showing potential in modulating microglial activity. Nevertheless, the complexity of neuroinflammation, which encompasses both protective and harmful responses, necessitates further research to fully unravel its role and optimize therapeutic approaches for AD.

## 1. Introduction

Alzheimer’s disease (AD) is the most prevalent cause of dementia, affecting millions globally, and one of the leading contributors to neurological disability and mortality [1]. The identification of accurate and early biomarkers for AD is a critical focus of current research, as early diagnosis and intervention are essential for altering the disease’s course and enhancing patient outcomes [2].

Neuropathologically, AD is characterized by the presence of extracellular senile plaques composed mainly of amyloid-β (Aβ) and intracellular neurofibrillary tangles composed of hyperphosphorylated tau protein [3]. The in vivo demonstration of altered biomarkers of Aβ pathology, tau deposition, and neurodegeneration constitutes the foundation of the ATN system, as outlined by the National Institute on Aging and the Alzheimer’s Association (NIA-AA) in 2018 [4]. The ATN system categorizes biomarkers into three groups: Aβ (A), tau pathology (T), and neurodegeneration or neuronal injury (N), which collectively represent the key pathological processes underlying AD [5]. The evolving ATN system was updated in 2024, with one of the important revisions being the inclusion of biomarkers for inflammatory processes, designated as “I”, highlighting the growing recognition of the role of neuroinflammation in AD [6].

Neuroinflammation, characterized by activation of the glial cells system, is a key component of AD etiology as supported by genetic, epigenetic, and proteomic studies [7]. Astrocytes and microglia are essential for the development and maintenance of neural circuits, with critical roles in synapse formation, neurotransmission, and brain homeostasis. In pathological conditions, they mediate inflammatory responses to amyloid plaques, tau tangles, and other stimuli [8,9]. While initially protective, chronic activation of these systems leads to the release of pro-inflammatory cytokines, disrupting normal functions and exacerbating neuronal injury [10,11]. Recent studies have shown that neuroinflammation is present from the earliest stages of AD, often preceding cognitive decline and overlapping with Aβ deposition [12].

The growing body of evidence pointing to the involvement of neuroinflammation in AD has led to an increased scientific interest in identifying biomarkers that reflect inflammatory processes in the brain [13]. Several neuroinflammatory fluid biomarkers, mainly related to microglial and astrocyte activity, have been identified as potential tools for the early detection and monitoring of AD progression. They include Glial Fibrillary Acidic Protein (GFAP), elevated in response to astrocyte reactivity; soluble Triggering Receptor Expressed on Myeloid Cells 2 (sTREM2), associated with microglial activation; and chitinase-3-like protein 1 (CHI3L1), also known as YKL-40 [14,15,16]. Furthermore, the development of blood-based biomarkers promises a non-invasive and longitudinal approach to assessing neuroinflammation in AD, making it easier to track disease progression and, possibly, therapeutic responses [2]. Advanced imaging techniques like positron emission tomography (PET) with tracers for TSPO and MAO-B provide insight into microglial and astrocyte activation [17,18]. These biomarkers offer significant potential for enhancing AD diagnosis and advancing our understanding of the disease’s neuroinflammatory mechanisms.

Despite advances in disease-modifying therapies for AD, such as monoclonal antibodies targeting Aβ, the clinical benefits still seem modest, and treatments are often associated with significant risks [19]. This has underscored the need for alternative therapeutic strategies that address other critical pathological mechanisms of AD, including neuroinflammation. Modulation of neuroinflammation is emerging as a key therapeutic strategy for slowing the progression of AD, with several candidates in both clinical and preclinical studies [20,21].

This review provides a detailed examination of the role of neuroinflammation in the pathogenesis of Alzheimer’s disease, highlighting key neuroinflammatory biomarkers and their potential application in transitioning research into clinical practice. Additionally, it explores the current therapeutic approaches and future research directions focused on targeting neuroinflammation in Alzheimer’s disease. By critically analyzing recent advancements, this review aims to summarize the state of the art in our understanding of neuroinflammatory processes in AD and assess how these insights can refine diagnostic methods and inform novel therapeutic strategies for this multifaceted disease.

## 2. Neuroinflammation: A Bridge Between Aβ Aggregation and Tau Tangle Accumulation?

In 1906, Alois Alzheimer first identified extracellular plaques and intraneuronal neurofibrillary tangles, along with significant glial reactions [22]. However, the role of immune processes, including microglial and astrocytic activity, only became a major research focus several decades later [20].

Astrocytes and microglia are essential for the development of neural circuits, exerting an important influence on synapses [23]. Astrocytes are vital for synapse formation and regulating neurotransmitter release, particularly maintaining glutamate balance in the brain [2]. Microglia, comprising 5–20% of all glial cells, act as the primary macrophages in the central nervous system (CNS). Their main role is to monitor the brain for pathogens and debris, while also protecting and remodeling synapses to maintain brain function [24,25,26]. Their roles are crucial for supporting synaptic plasticity and cognitive function.

AD pathology is characterized by the presence of extracellular amyloid plaques composed of Aβ and intracellular neurofibrillary tangles (NFTs) made of hyperphosphorylated tau protein [3].

Aβ plaques can form years before clinical symptoms of AD emerge [27,28]. These toxic Aβ aggregates are recognized by the immune system as danger-associated molecular patterns (DAMPs), which are detected by microglia through various receptors, including the receptor for advanced glycation end products (RAGE), nucleotide-binding oligomerization domain-like receptors (NLRs), and Toll-like receptors (TLRs) [29,30]. These surface receptors can detect various substrates, including bacterial or viral products, DNA, and neurodegenerative proteins [3]. This recognition triggers microglial activation, leading to an initial protective response aimed at clearing the harmful aggregates. Astrocytes also play a critical role in the response to Aβ plaques, surrounding them to isolate the pathological deposits from healthy brain tissue [31,32]. However, when Aβ clearance fails, chronic activation of astrocytes occurs, leading to a pathological state. In this state, pro-inflammatory cytokines such as interleukin-1β (IL-1β), tumor necrosis factor-alpha (TNF-α), and interleukin-6 (IL-6) are released, which contribute to neuronal injury and further propagate inflammation [20,33]. This chronic inflammation disrupts normal astrocytic functions, including phagocytosis and metabolic support, thereby exacerbating the disease process [34].

Under physiological conditions, tau protein is essential for regulating microtubule dynamics, glucose metabolism, and other cellular functions [3,35]. In AD, however, tau becomes hyperphosphorylated by specific kinases such as glycogen synthase kinase-3β (GSK-3β) and cyclin-dependent kinase 5 (CDK5) [36]. The activity of these kinases may be promoted by pro-inflammatory cytokines [37]. Hyperphosphorylated tau loses its ability to bind to microtubules, leading to its detachment and subsequent aggregation within neurons and in the extracellular space [38,39]. These toxic tau aggregates spread through the brain in a prion-like manner, disrupting essential cellular functions and contributing significantly to neurodegeneration [39]. Reactive microglia are often observed near NFTs, with pathological tau believed to act as a DAMP, further activating microglia and triggering an immune response [40,41,42]. This could contribute to the chronic inflammation observed in AD. The interaction between Aβ and tau pathologies within the inflammatory environment exacerbates the progression of the disease, creating a vicious cycle of neurodegeneration [43]. DAMPs released from dying neurons, such as ATP, S100B, and DNA, further amplify inflammation, establishing a positive feedback loop [44,45].

In summary, the inflammatory environment created by persistent Aβ accumulation is thought to facilitate the transition from Aβ plaque deposition to tau pathology [46,47]. As the disease progresses, the sustained activation of microglia and astrocytes contributes to the breakdown of the blood–brain barrier, further facilitating the infiltration of peripheral immune cells into the CNS [48,49]. This exacerbates the local inflammatory environment and accelerates neuronal damage. In addition, chronic inflammation in AD is also linked to the activation of inflammasomes, particularly the NLRP3 inflammasome, which plays a critical role in promoting tau hyperphosphorylation and aggregation, further driving the disease process [50,51]. Moreover, the role of astrocytes and microglia in AD extends beyond their inflammatory response [52,53]. These cells are involved in the regulation of synaptic function and the maintenance of neuronal health [54]. Their prolonged activation can lead to the release of neurotoxic substances that impair synaptic function and promote neuronal death [55]. This pathological crosstalk between glial cells and neurons highlights the complex and multifaceted nature of neuroinflammation in AD.

**Figure 1:**
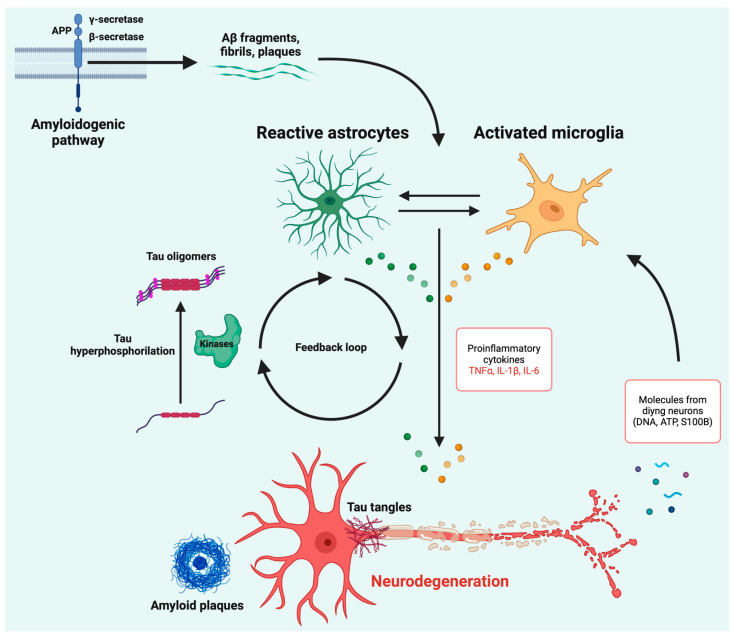
Aβ fibrils and plaques can trigger an immune response through microglial receptors. This activates microglia and astrocytes, which attempt to clear the plaques. However, chronic inflammation occurs when clearance fails, leading to the release of pro-inflammatory cytokines like IL-1β, TNF-α, and IL-6, which contribute to neuronal damage. Concurrently, pro-inflammatory cytokines promote the activity of kinases involved in the hyperphosphorylation of tau. Tau proteins become hyperphosphorylated, forming toxic aggregates that spread and worsen neurodegeneration, with molecules released from dying neurons that further amplify neuroinflammation. The interaction between Aβ and tau in an inflammatory environment drives AD progression, creating a vicious cycle of damage. Created in Biorender.com. Roveta, F. (2024) https://BioRender.com/t93c382.

Figure 1 summarizes the mechanisms of neuroinflammation that have been implicated in the pathogenesis of Alzheimer’s disease.

## 3. Current Evidence on Neuroinflammatory Biomarkers of Alzheimer’s Disease

Neuroinflammation plays a crucial role in AD pathogenesis, with recent studies highlighting several neuroinflammatory biomarkers that provide insight into the disease’s mechanisms and diagnosis. These biomarkers, mainly based on microglial cells and astrocytes, offer potential for the early detection and understanding of AD. In this section, we highlight key neuroinflammatory biomarkers, both fluid- and PET-based, emphasizing their clinical relevance and potential implications. Table 1, at the end of this summary, provides an overview of the most extensively studied biomarkers in Alzheimer’s disease neuroinflammation, outlining their main characteristics.

### 3.1. Glial Fibrillary Acidic Protein (GFAP)

In Alzheimer’s disease, astrocytes adopt a reactive phenotype, with upregulation of proteins such as GFAP, a key cytoskeletal component [56]. In AD, astrocytes respond to both neurofibrillary tangles and Aβ plaques, with effects that can be either neuroprotective or harmful [57]. GFAP levels are elevated in the CSF and plasma of AD patients [14,58]. Higher GFAP concentrations are linked to Aβ plaque density and white matter injury, correlating with cognitive decline [59,60]. Plasma GFAP, especially when combined with other biomarkers like the Aβ1-42/Aβ1-40 ratio and APOE ε4 status, has shown promise in improving diagnostic accuracy in AD [61]. Longitudinal studies have highlighted plasma GFAP as a biomarker for early detection [62]. Increased GFAP levels have been detected up to a decade before the onset of AD symptoms in individuals at risk. Studies also show that higher baseline GFAP concentrations are associated with the progression of MCI to AD and faster cognitive decline [63,64]. In addition, GFAP has demonstrated predictive value for dementia risk, as higher plasma levels have been linked to increased dementia incidence and mortality in several long-term studies [61,65]. Compared to other biomarkers like NfL, GFAP may serve as a more effective early indicator of astrocyte reactivity and neurodegeneration in preclinical AD, making it a promising tool for identifying individuals at risk before clinical symptoms emerge [61,66].

### 3.2. Soluble Triggering Receptor Expressed on Myeloid Cells 2 (sTREM2)

TREM2, a receptor primarily expressed on microglia, plays a key role in enhancing microglial and macrophage phagocytosis and modulating inflammatory signaling [67]. TREM2 can bind to various ligands, including Aβ, initiating downstream signaling cascades that promote the survival, proliferation, and phagocytic activity of microglia [68,69]. Mutations in the *TREM2* gene have been associated with an increased risk of developing late-onset Alzheimer’s disease [70], probably through a reduced microglial ability to clear Aβ, impairing the inflammatory response [15]. A soluble form of TREM2 (sTREM2) is produced by proteolytic cleavage from the cell surface, contributing to microglial activation [71]. Although some studies indicate higher levels of sTREM2 in the CSF of patients with AD and MCI [72,73], its diagnostic power seems limited [2]. In individuals with autosomal dominant AD, CSF sTREM2 concentrations begin to rise approximately five years before the onset of symptoms, but after the accumulation of Aβ plaques [74]. Studies have shown that CSF sTREM2 levels are inversely related to Aβ pathology, with lower sTREM2 concentrations linked to increased amyloid burden in the brain [75]. Conversely, higher sTREM2 levels are associated with tau-related neurodegeneration, suggesting that sTREM2 could serve as an indicator of the transition from amyloid-driven pathology to tau-mediated neurodegeneration [76]. Elevated sTREM2 levels are linked to brain structural changes, suggesting its role in neuroinflammatory responses during early neurodegeneration.

### 3.3. Chitinase-3-like Protein 1 (YKL-40)

YKL-40, is produced by various cells, particularly reactive astrocytes [77]. While its function is not fully understood, it plays a role in tissue remodeling, inflammation, and angiogenesis, making it a potential biomarker for neuroinflammation [78]. YKL-40 is closely associated with glial activation, including both astrocytes and microglia, and has been associated with AD tau pathology [16,79]. Studies have shown that CSF levels of YKL-40 are elevated in AD and MCI patients, often correlating with markers of neurodegeneration, such as t-tau, and synaptic damage [80]. Its increased levels in CSF can help differentiate dementia patients from cognitively unimpaired individuals and predict cognitive decline, particularly in those transitioning from preclinical to more advanced stages of AD [81]. Plasma YKL-40 levels have also been shown to be higher in early AD and MCI cases compared to controls, with associations found between YKL-40 levels and cognitive performance [82]. Additionally, YKL-40 levels may vary based on factors such as sex and ethnicity [83,84]. Its value as a biomarker comes from its ability to reflect glial activation and inflammation, making it useful for tracking disease progression and informing clinical trials targeting neuroinflammatory pathways [83,85,86]. Elevated YKL-40 in CSF, even in asymptomatic individuals, could be an early marker of neurodegeneration [2,16].

### 3.4. S100B

S100B is primarily expressed in astrocytes and plays a neurotrophic role under normal conditions [56,87]. It is also found in non-neuronal cells, limiting the use of blood measurements as a direct marker of brain pathology, and CSF S100B levels are considered more reliable for detecting reactive astrogliosis [88]. However, studies of CSF S100B in Alzheimer’s disease have produced mixed results. Some research has found a moderate increase in early AD [89,90], while other research did not detect significant differences from controls [91,92]. No associations between S100B and Aβ levels have been observed [93,94], and increased S100B in CSF is found in other neurodegenerative diseases as well, such as Parkinson’s and Lewy body dementia [56,88].

### 3.5. 18 kDa Translocator Protein (TSPO)

TSPO, the most studied microglial PET target, is expressed in the outer mitochondrial membrane of microglia, astrocytes, and endothelial cells [85,95]. It is upregulated by microglial cells under pathological conditions, making TSPO-PET a common tool for detecting microglial activation [17]. TSPO-PET signals have shown increased activity in AD-related brain regions, such as the hippocampus and cortex, as seen with the first TSPO tracer, [^11^C]-(R)PK11195 [96]. However, second-generation TSPO tracers like [^11^C]-DPA713 and [^11^C]-DAA1106 [97] have shown varied results, and genetic polymorphisms in the TSPO gene (rs6971) affecting binding affinity require genetic testing for accuracy [98]. Newer third-generation tracers, such as [^18^F]GE-180, are being developed to avoid this issue and have shown increased PET signals in the cortical regions of AD patients [99]. Despite their widespread use, TSPO tracers have some limitations due to their lack of specificity for microglia.

### 3.6. Monoamine Oxidase B (MAO-B)

The first PET tracer for astrocytes was developed for MAO-B, an enzyme located in the outer mitochondrial membrane [100]. MAO-B is mainly found in astrocytes and becomes upregulated in reactive astrocytes [101,102]. Initial studies using the radiotracer [^11^C]-DED revealed increased MAO-B activity in AD patients, particularly in their cortical and hippocampal regions [18,103]. Later research suggested that this increase occurs earlier in the disease, peaking during prodromal AD stages [104]. Studies of [^11^C]-DED-PET found increased binding in Aβ-positive individuals with MCI and carriers of autosomal dominant AD mutations, with binding decreasing with increasing amyloid load [105]. Other MAO-B radiotracers have been developed, showing promising results in detecting astrocyte reactivity across the AD continuum [56]. [^18^F]-SMBT-1 has been investigated in MCI and AD patients, showing increased uptake in cortical brain regions in Aβ-positive individuals [106].

### 3.7. Other Neuroinflammatory Biomarkers

Other inflammatory biomarkers, such as cytokines, chemokines, and growth factors, are not disease-specific but have potential as neuroinflammatory markers in Alzheimer’s disease. Cytokines, like IL-6, IL-1β, and TGF-β, are found near Aβ plaques but reflect general immune alterations [107]. Several peripheral factors affect their levels, making their diagnostic use limited. Additionally, cytokines such as basic fibroblast growth factor (bFGF), CRP, IL-16, and VEGF-D, among others, are measurable in serum and CSF [2,108]. Studies show that adding these biomarkers to traditional AD markers could improve diagnostic accuracy in cognitively impaired individuals [2,56]. However, inconsistent results and the lack of longitudinal studies hinder their clinical application. Further research is needed to confirm their prognostic value in AD.

Among new PET imaging targets for microglia, the Colony-Stimulating Factor-1 Receptor (CSF1R), expressed by microglia and macrophages, appears to be promising. Preclinical studies using tracers such as [^11^C]-CPPC have detected increased binding in mouse models of amyloid pathology, indicating its ability to capture microglial changes [109]. However, no PET studies have been conducted in humans, although postmortem analyses have shown increased expression of CSF1R in AD brains [110]. CB2R, part of the endocannabinoid system, is upregulated in microglia during immune activation [56]. A recent study showed lower CB2R availability in AD mouse models, but further exploration is needed [111]. Other potential targets for microglia in AD include TREM1 and TREM2. They have shown potential in preclinical studies, but clinical PET studies targeting these receptors have not yet been conducted [56]. A potential target for astrocytes is I2-BS, which is located in the outer mitochondrial membrane and has been studied with the tracer [^11^C]-BU99008, as it has shown higher specific binding in AD brains, particularly in MCI patients [112,113,114]. Astrocyte metabolism can also be imaged using markers like [^11^C]-acetate, which has shown increased uptake in AD-vulnerable regions such as the medial temporal lobe and hippocampus in Aβ-positive MCI patients [115,116].

**Table 1:** Summary of neuroinflammation biomarkers involved in Alzheimer’s disease.

Biomarker/Target	Category	Role/Mechanism	Clinical Evidence	Ref.
GFAP	CSF/Blood	Marker of reactive astrocytes.	Increased CSF and plasma levels in AD, linked to Aβ pathology and AD progression. Plasma GFAP is stable and predictive of conversion from MCI to AD dementia.	[14,58,63,64]
sTREM2	CSF	Released during microglial activation through TREM2 shedding.	Elevated in CSF with AD progression; correlates with higher tau pathology and slower cognitive decline. Conflicting evidence regarding levels between AD patients and cognitively unimpaired individuals.	[72,73,74,75,76]
YKL-40	CSF	Expressed in reactive astrocytes and microglia during neuroinflammation.	Elevated in CSF during later AD stages; associated with Aβ and tau pathology, brain atrophy, and cognitive decline. Rises earlier in familial AD.	[80,81,82]
S100B	CSF	Associated with neuroinflammation.	Inconsistent evidence; some studies show moderate increases in early stages, while others show no significant differences. Expression in other tissues complicates interpretation.	[88,89,90,91,92]
TSPO	PET	Upregulated under pathological conditions; detected via PET imaging.	Increased TSPO binding occurs in AD-affected brain regions, but lack of specificity for microglia creates limitation. Newer tracers under development.	[17,96,98]
MAO-B	PET	Enzyme localized in astrocytes, detectable via PET imaging.	Elevated activity in AD patients’ temporal cortex and hippocampus, particularly in early stages. Activation peaks during MCI stage.	[18,103,104]

Abbreviations: GFAP, Glial Fibrillary Acidic Protein; sTREM2, Soluble Triggering Receptor Expressed on Myeloid Cells 2; YKL-40, Chitinase-3-like Protein 1; S100B, S100 Calcium-Binding Protein B; TSPO, 18 kDa Translocator Protein; MAO-B, Monoamine Oxidase B.

## 4. Therapeutic Strategies

Modulating neuroinflammation has garnered considerable attention as a promising therapeutic strategy to slow the progression of AD. Numerous studies are currently exploring therapies aimed at ameliorating neuroinflammatory pathways, with some of the most advanced candidates in the pipeline outlined below.

As discussed in previous sections, TREM2 is a receptor expressed on microglial cells that plays a key role in modulating their response to Aβ, and it is the focus of several studies exploring its potential as a therapeutic target. Preclinical studies have demonstrated that the activation of TREM2 enhances microglial survival and phagocytic activity, leading to a reduction in amyloid burden and neuroinflammation [117]. Clinical trials are currently investigating both monoclonal antibodies and small-molecule agonists designed to target TREM2, with the aim of translating these promising preclinical findings into effective treatments for Alzheimer’s disease. Among them, the trial NCT04592874 aims to evaluate the safety, tolerability, and efficacy of AL002, a monoclonal antibody targeting TREM2 in patients with early AD, with the goal of enhancing microglial function to reduce neuroinflammation and possibly slow disease progression [118]. Another possible approach to modulating microglial activity and neuroinflammation could be represented by the inhibition of CSF1R, which presents a promising strategy to attenuate neuroinflammation by reducing microglial proliferation and altering their inflammatory phenotype [119]. This approach shows potential in slowing Alzheimer’s pathology progression and improving cognitive outcomes in preclinical models [120].

Antidiabetic drugs, particularly metformin and GLP-1 receptor agonists like liraglutide and semaglutide, are being explored as potential treatments for neuroinflammation in AD [121]. Metformin activates AMP-activated protein kinase, which inhibits the NF-κB signaling pathway, reducing the production of pro-inflammatory cytokines such as TNF-α, IL-1β, and IL-6 [122]. This action helps mitigate neuroinflammation and promotes the clearance of Aβ. Clinical trials have shown that metformin improves cognitive functions like memory and attention in AD patients, indicating its potential therapeutic benefit [123,124]. GLP-1 receptor agonists, commonly used for type 2 diabetes and obesity, have shown neuroprotective properties by modulating microglial activity, reducing oxidative stress, and enhancing neuronal survival [125]. A case–control study involving 176,250 individuals with type 2 diabetes revealed a substantial reduction in the incidence of dementia among those treated with GLP-1 receptor agonists [126,127]. Currently, two phase III randomized, double-blind, placebo-controlled clinical trials (NCT04777396 and NCT04777409) to evaluate safety and efficacy of semaglutide in AD patients are ongoing [128].

Growing evidence highlights the role of gut–brain axis dysregulation in exacerbating neuroinflammation [3,129]. Strategies aimed at normalizing gut microbiota, such as the use of probiotics, prebiotics, or fecal transplantation, may offer a valuable tool in AD prevention or treatment [130].

Lastly, studies have highlighted that targeting epigenetic mechanisms, such as DNA methylation and histone modifications, could represent a novel therapeutic strategy to counteract neuroinflammation in AD [131]. By modulating the expression of inflammatory genes, these approaches may help mitigate the disease’s progression, adding a complementary dimension to current anti-inflammatory and neuroprotective treatments [132,133].

## 5. Discussion and Conclusions

The growing recognition of neuroinflammation as a central element in Alzheimer’s disease pathogenesis has expanded the scope of research beyond traditional amyloid-β and tau pathology, offering new directions for both diagnostic biomarkers and therapeutic interventions. The inclusion of the category of inflammatory biomarkers in the ATN system underscores the importance of neuroinflammation in AD progression and reflects a shift toward understanding the broader mechanisms involved [6].

Recent evidence highlights that neuroinflammation, characterized by the chronic activation of microglia and astrocytes, is present even in the earliest stages of AD. This suggests that inflammatory processes may not only exacerbate, but also initiate key pathological events, such as the deposition of Aβ plaques and the formation of neurofibrillary tangles [44,45]. The interaction between Aβ and tau within a neuroinflammatory environment creates a vicious cycle of neuronal damage and further inflammation [43], suggesting that targeting inflammation could break this cycle and slow disease progression.

Several neuroinflammatory biomarkers, including GFAP, sTREM2, and YKL-40, have emerged as promising tools for early detection and disease monitoring [2]. Plasma GFAP has shown utility in predicting the conversion from MCI to AD dementia, offering a potential non-invasive biomarker for preclinical disease stages. Importantly, longitudinal studies have shown that higher baseline GFAP concentrations are associated with faster cognitive decline and increased risk of dementia, further highlighting its utility in early disease monitoring [61,65]. The recent proposal to expand the ATN system to incorporate GFAP into this framework could offer a more comprehensive view of the disease process, especially in early stages [6]. This proposal aligns with the growing evidence of astrocyte involvement in AD, with GFAP serving as a marker of astrocyte reactivity.

Similarly, sTREM2 has shown promise as a marker of microglial activation, though its diagnostic utility appears to be more closely associated with tau-related neurodegeneration rather than early amyloid pathology [72,73,75]. Understanding its role is complicated by mixed findings in longitudinal studies. While some research links elevated CSF sTREM2 levels to an increased risk of clinical dementia conversion, other studies suggest that higher sTREM2 levels may be protective, correlating with slower cognitive decline [56]. This inconsistency may reflect the evolving role of microglia at different stages of the disease. Therefore, future research must focus on the temporal dynamics of neuroinflammatory biomarkers, exploring their distinct patterns across the Alzheimer’s disease continuum. The wider use of sTREM2 is limited by the fact that it is not currently easily measurable in blood.

The development of advanced imaging techniques, such as PET tracers targeting TSPO and MAO-B, has further enriched our understanding of glial activation in AD. These imaging biomarkers provide new insights into the spatial and temporal dynamics of neuroinflammation, which could be crucial for tailoring therapeutic strategies [106]. TSPO-PET imaging allows the visualization of microglial activation in vivo, offering valuable topographical information on the distribution and progression of neuroinflammation in AD [85,95]. Although TSPO tracers have limitations due to their lack of specificity for microglia and genetic variability in TSPO expression, the ongoing development of third-generation tracers seeks to address these issues.

Several therapeutic approaches targeting neuroinflammation are currently under investigation. TREM2-targeting therapies, such as the monoclonal antibody AL002, show promise in modulating microglial activity to reduce amyloid burden and inflammation [117]. Antidiabetic drugs like metformin and GLP-1 receptor agonists are being repurposed for AD due to their anti-inflammatory and neuroprotective effects [128]. Clinical trials evaluating these agents, particularly semaglutide, represent an exciting frontier in the development of novel AD treatments. In addition to these pharmacological approaches, growing evidence highlights the potential of modulating the gut–brain axis [130].

Despite these advances, translating neuroinflammatory insights into clinical practice remains a significant challenge. One key issue is the lack of specificity of these biomarkers, which can be elevated in multiple conditions beyond AD [12]. Moreover, the precise context of the use of these biomarkers is not yet fully clarified and, at present, they are still primarily designated for research use. With the advent of new treatments for Alzheimer’s disease, these biomarkers hold significant potential for integration into clinical practice, enabling more effective monitoring of disease progression and therapeutic response [2]. Future efforts should focus on combining multiple inflammatory biomarkers to enhance specificity and provide a more accurate reflection of the disease’s state.

The dual nature of neuroinflammation in Alzheimer’s disease, encompassing both protective and harmful responses, necessitates a deeper understanding of its underlying pathophysiological mechanisms. For instance, while the activation of microglial cells can initially be neuroprotective by facilitating the clearance of Aβ, chronic microglial activation leads to a detrimental inflammatory response that exacerbates neuronal damage. Therefore, therapeutic strategies should aim to balance these responses, enhancing protective functions while mitigating chronic inflammation. Further research is essential to unravel these complexities and guide the development of targeted and effective therapeutic strategies.

In conclusion, the role of neuroinflammation in AD has become a critical focus of both research and clinical investigation. While progress has been made in identifying biomarkers and therapeutic targets, much remains to be understood about the complex and multifaceted nature of neuroinflammation in AD. Future research should prioritize refining these biomarkers to ensure their clinical utility and appropriate context of use. The ultimate goal is to enhance diagnostic and prognostic accuracy while effectively monitoring therapeutic interventions as they emerge.

## 6. Search Strategy and Inclusion Criteria

This non-systematic review provides an overview of the current evidence on neuroinflammation biomarkers in Alzheimer’s disease. The manuscript is based on a selective analysis of recent high-quality articles on neuroinflammation biomarkers in Alzheimer’s disease. The main objective is to highlight trends and improve understanding of the current biomarker landscape in this context. Relevant references were sourced from PubMed, the Web of Science, or Scopus. Search terms included “Biomarker”, “Neuroinflammation”, and “Alzheimer”. Additional articles were identified by reviewing the bibliographies of relevant papers, with only English-language studies included.
